# A Novel Highly Conductive, Transparent, and Strong Pure-Cellulose Film from TEMPO-Oxidized Bacterial Cellulose by Increasing Sonication Power

**DOI:** 10.3390/polym15030643

**Published:** 2023-01-26

**Authors:** Dieter Rahmadiawan, Hairul Abral, Rafi Alzues Kotodeli, Eni Sugiarti, Ahmad Novi Muslimin, Ratna Isnanita Admi, Andril Arafat, Hyun-Joong Kim, S.M. Sapuan, Engkos Achmad Kosasih

**Affiliations:** 1 Department of Mechanical Engineering, Universitas Negeri Padang, Padang 25173, Indonesia; 2 Laboratory of Nanoscience and Technology, Department of Mechanical Engineering, Andalas University, Padang 25163, Indonesia; 3 Research Collaboration Center for Nanocellulose, BRIN-Andalas University, Padang 25163, Indonesia; 4 Laboratory of High-Temperature Coating, Research Center for Physics, Indonesian Institute of Sciences (LIPI) Serpong, Banten 15314, Indonesia; 5 Faculty of Defense Technology, Indonesia Defense University, Bogor 16810, Indonesia; 6 Laboratory of Adhesion & Bio-Composites, Program in Environmental Materials Science, Research Institute for Agriculture & Life Sciences, Seoul National University, Seoul 151-921, Republic of Korea; 7 Advanced Engineering Materials and Composites Research Centre (AEMC), Department of Mechanical and Manufacturing Engineering, Universiti Putra Malaysia (UPM), Serdang 43400, Malaysia; 8 Department of Mechanical Engineering, Faculty of Engineering, Kampus UI, Universitas Indonesia, Depok 16424, Indonesia

**Keywords:** bacterial cellulose, electrical conductivity, thermal resistance, ultrasonication treatment

## Abstract

Developing a conductive cellulose film without any metal compounds remains challenging, though in great demand. However, cellulose film prepared from bacterial cellulose (BC) powder without any metal compounds has poor tensile, physical, and electrical properties, thus limiting its application. Herein, this study aims to prepare and characterize an all-cellulose film from 2,2,6,6-Tetramethylpiperidin-1-yl)oxyl (TEMPO)-oxidized bacterial cellulose (TOBC) powders without adding metal compounds and treated by ultrasonication. TOBC powders are sonicated with various powers of 250, 500, and 750 W for 20 min without any other substance. It was proved that increasing the ultrasonication power level resulted in a significant improvement in the properties of the film. The ultrasonication of 750 W increased tensile strength by 85%, toughness by 308%, light transmittance by 542%, and electrical conductivity by 174% compared to the nonsonicated film. A light-emitting diode connected to a power source through this sonicated film was much brighter than that connected via a nonsonicated film. For the first time, this study reports the preparation of electrically conductive, transparent, strong, and bendable pure TOBC films by increasing ultrasonic power for environmentally friendly electronic devices application.

## 1. Introduction

There are many sources of nontoxic material in the world [1,2]. Cellulose is an abundant, inexpensive, and readily available carbohydrate polymer with many advantages: biocompatibility with the human body, biological degradability, and high specific strength and modulus [3]. Naturally sourced cellulose has been used as a filler in biocomposites to improve the mechanical and thermal properties of biofilms [4,5] and as a base material for strong, flexible, and conductive films [6,7,8,9]. It can be obtained from plants such as cotton [10], kenaf [11], ginger [12], or rice straw [13], but the isolation process generally uses harsh chemicals, thus causing environmental pollution.

Various bacteria can also be used as a cellulose source. For instance, the wet pellicle of *Acetobacter Xylinum* bacterium is a rich source of pure cellulose that has an equivalent chemical structure to plant cellulose [14]. The manufacturing of BC pellicle is simple, inexpensive, and does not require environmentally damaging chemicals [15]. When reduced to nanofibres, BC has excellent mechanical and thermal properties, high purity and crystallinity, and a large specific surface area, enabling it to have many potential applications [16,17,18].

Films manufactured using the dry BC powder only, without further processing, have low tensile properties. For electronic device purposes, BC has poor performance due to its insulating nature. This can be overcome by adding nanoparticles, mostly made of metal, that can improve the electrical performance of BC properly. Zhang et al. [19] developed a conductive BC film by adding graphene oxide. The results showed that graphene could provide conductivity in the range of 0.001 to 12 S/m, depending on the graphene content. Further, a low concentration of multiwall carbon nanotubes (0.0041 volume concentration) cooperated with a BC film, with an electrical conductivity of 0.01 S/m [20].

However, metal nanoparticles are poisons and nonrenewable materials, which also requires many steps to synthesize in fine quality [21]. This limitation can be overcome by using ultrasonication [22,23]. By conducting ultrasonication treatment, the tensile strength of the BC powder-based film is increased significantly. Ultrasound energy from cavitation (the formation, growth, and violent collapse of cavities in water) is transferred to cellulose chains [24]. This ultrasonic impact can gradually disintegrate the micron-sized cellulose fibers into nanofibers. Hence, ultrasonication reduces the cellulose fiber size. It also increases the transparency of the cellulose film [25].

TEMPO treatment oxidizes BC nanofibers and weakens BC’s hydrogen bonds, presenting better dispersibility [26,27,28,29,30]. This treatment can promote individual nanofibers and a nanogap between BC nanofibers. Recently, Huang et al. [31] developed conductive TEMPO-oxidized cellulose films from wet BC pellicles fibrillated into a slurry by using a high-speed homogenizer. However, this conductive film was not fully transparent. We hypothesized that a pure BC film made from TEMPO-oxidized bacterial cellulose powders would be even more electrically conductive, transparent, strong, and bendable if the powder was first sonicated. The reason is that the ultrasonication of the BC cellulose can add free hydroxyl as an electron-donating functional group [32,33]. As far as the authors are aware, there have been no published studies developing these pure TOBC films without any additional metal compounds to increase the electrical conductivity of the film by using this method. Therefore, the present work was prepared this way and demonstrated that the process improved the pure TOBC film properties.

## 2. Materials and Methods

### 2.1. Materials

We used wet BC pellicle cuboids (350 × 250 × 5 mm) similar to those used in our previous work that were obtained from a small-scale industry in Padang, Indonesia, in the form of nata de coco. 2,2,6,6-Tetramethylpiperidine-1-oxyl (TEMPO), sodium hypochlorite (NaClO), sodium hydroxide (NaOH), and sodium bromide (NaBr) were purchased from Sigma-Aldrich Co, Burlington, MA, USA.

### 2.2. Preparation of TEMPO-Treated BC Film

**Figure 1:**
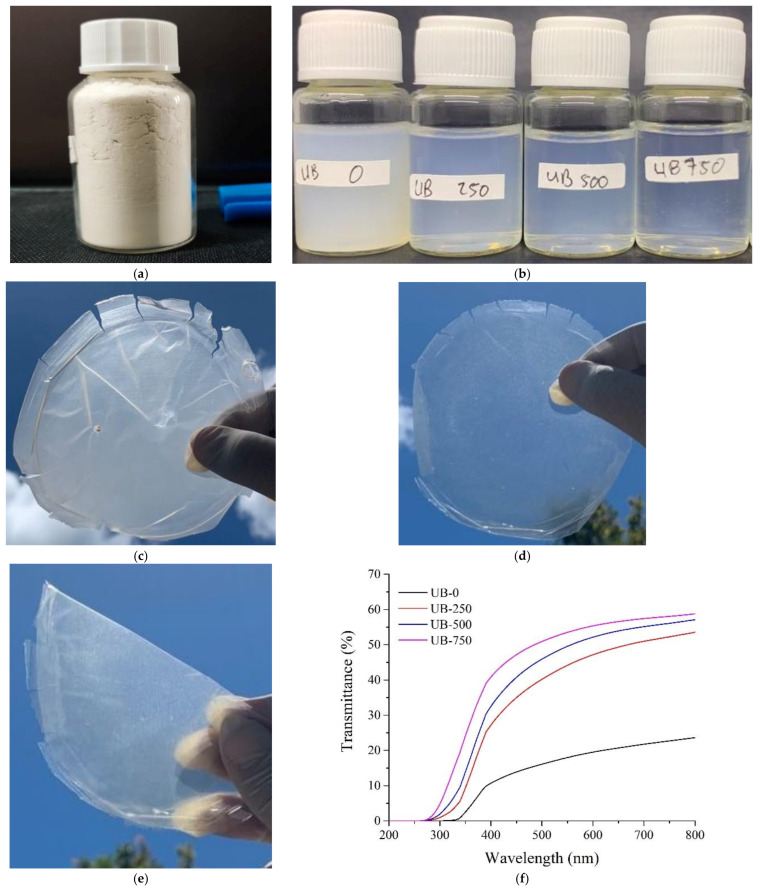
(**a**) BC powder, (**b**) appearance of treated BC suspension from various ultrasound energy, (**c**) translucent UB-0 film, (**d**) transparent UB-750 film, (**e**) bendable UB-750 film, (**f**) transmittance value of films.

*Preparation of BC powder*: A wet pellicle was treated using 10% NaOH solution for 48 h to obtain pH 10. Next, a conventional blender disintegrated the wet pellicle at 12,000 rpm for one hour. It was dried using a Universal Oven Memmert UN-55 drying oven at 70 °C for 24 h. The dried pellicle was crushed using a CE-High-Speed Multifunctional grinder at 3600 rpm for one hour and was followed by filtration using 60, 100, or 200 mesh sieves to obtain BC powder (Figure 1a).

*Preparation of TOBC suspension*: A total of 1 g of BC powder (200 mesh) was added into 200 g of distilled water (0.5 *wt*%), 0.018 g of TEMPO, 0.2 g of NaBr, and 7.4 g of NaClO [31]. The mixture was stirred using a hot plate magnetic stirrer (Daihan MSH-20D) at 50 °C for 20 min to oxidize the BC powder. A NaOH (0.5 M) solution was added to the TEMPO-oxidized BC powder (TOBC) suspension until the pH reached 10, and then the suspension was heated using the Daihan MSH-20D stirrer at 70 °C for 6 h. It was left to cool naturally for 12 h. The TOBC suspension was placed in four different beakers for ultrasonication treatment at 0 W, 250 W, 500 W, or 750 W power.

*Preparation of TOBC film*: The TOBC suspensions were poured into a glass tube for centrifugation using an LD-3 Electronic Centrifuge machine for 30 min at 3000 rpm. After the centrifuge, TOBC nanofiber sediment was evident at the bottom of the tube. The water in the tube was removed and replaced with fresh distilled water. This process was repeated until the suspension was pH 7. Then, the residue was treated using a high-shear homogenizer (WiseTis Homogenizer HG-15D DAIHAN Scientific Co., Ltd. from Gangwon-do, Korea) at 8000 rpm for 30 min. It was then treated using an FS-1200N ultrasonication homogenizer according to different powers (250, 500, and 750 W) for 20 min. Each sonicated BC suspension was heated using a magnetic stirrer at 100 °C for 4 h. After it had cooled to room temperature, it was poured on a Teflon plate (12 cm diameter) for drying using a Universal Oven Memmert UN-55 drying oven at 50 °C for 48 h. The dry TEMPO-oxidized BC films were stored in a vacuum desiccator with RH 50%. We marked nonsonicated samples with UB-0 film and the 250 W, 500 W, and 750 W ultrasonicated films as UB-250, UB-500, and UB-750, respectively.

## 3. Characterization

### 3.1. Field Emission Scanning Electron Microscopy (FESEM) Observation

A FESEM (JFIB-4610, JEOL, Akishima, Japan), was used to observe the surface morphology of the samples. An accelerating current of 15 kV and probe current of 8 mA were selected to optimize the observation. The BC film samples were placed on the FESEM sample stub. The sheet was coated with carbon followed by gold to reduce the electron charge.

### 3.2. Film Transmittance

The transmittance of films was measured using a UV/Visible Scanning Spectrophotometer, UV-1800 (Shimadzu, Kyoto, Japan). Rectangular samples (1 × 2.5 cm) were prepared and placed in the spectrophotometer. The spectrum used was between 200 and 800 nm. Transmittance determination was repeated three times.

### 3.3. Fourier-Transform Infrared Spectroscopy (FTIR)

FTIR of nonsonicated and sonicated BC samples were characterized using Frontier IR equipment (PerkinElmer, Waltham, MA, USA). The dried samples were formed into a sheet film and scanned at a frequency range of 4000–400 cm^−1^ at 4 cm^−1^ resolution.

### 3.4. Thermal Resistance

The thermal resistance of samples was measured using a thermal analysis apparatus, DTG-60 (Shimadzu, Kyoto, Japan). The sample was then placed in the instrument with a 50 mL/min nitrogen flow rate. The temperature increased at a pace of 20 °C/min.

### 3.5. Tensile Properties

Tensile properties of the samples were measured using COM-TEN 95T Series 5K (Pinellas Park, FL, USA) using a tensile test speed of 5 mm/min at room temperature according to the ASTM D 638 type V standard. Before the test, all samples were stored in a desiccator with a relative humidity of 50% at 25 °C for 48 h. Tensile tests were repeated five times for each treatment.

### 3.6. X-Ray Diffraction

X-ray diffraction testing was performed using a X-ray diffractometer (PANalytical X’pert pro, Amsterdam, The Netherlands) at 25 °C, 40 kV, and 30 mA. The samples were scanned from 2θ = 3° to 90°. Normally, the BC derived from *Acetobacter Xylinum* is dominant in cellulose Iα [34]. Therefore, the X-ray diffraction patterns will be labeled according to cellulose Iα [35]. The crystallinity index (CI) was measured using Equation (1) [36]:

(1) $$CI\;\left(\%\right)=\frac{\left(I_{110}-I_{am}\right)}{I_{110}}\times 100$$

where *I*_110_ is the maximum intensity of the peak (2θ = 22.6°) and *I_am_* is the minimum intensity of the amorphous fraction at about 2θ = 18.7°.

### 3.7. Electrical Properties

Samples were stored in a vacuumed desiccator with RH 50%. Their electrical resistance (*R*) was measured using a M-3 portable handheld four-point probe tester (Suzhou Jingge Electronic Co., Ltd., Suzhou, China) at room temperature. Resistance measurements were carried out five times on the sample surface in the same direction and averaged to ensure data accuracy. The resistivity and conductivity values were calculated by Equations (2) and (3), respectively:

(2) $$\rho =\frac{\pi t}{ln2}R$$

(3) $$\sigma =\frac{1}{\rho }$$

where *ρ* is the resistivity (Ω cm), *σ* is the conductivity (S/cm), *t* is the film thickness (mm), and *R* is the resistance (Ω) of films.

### 3.8. Statistical Analysis

IBM SPSS Statistics 25.0 (IBM Corporation, Chicago, IL, USA) was used to analyze the experimental data. A one-way analysis of variance (ANOVA) and *p*-test were carried out to identify the significance of the effect of each treatment effect on the film properties. Duncan’s multiple range test was used with a 95% confidence level (*p* ≤ 0.05).

## 4. Results and Discussions

### 4.1. BC Powder, Suspension, a Film Appearance, and Transmittance Value

**Figure 2:**
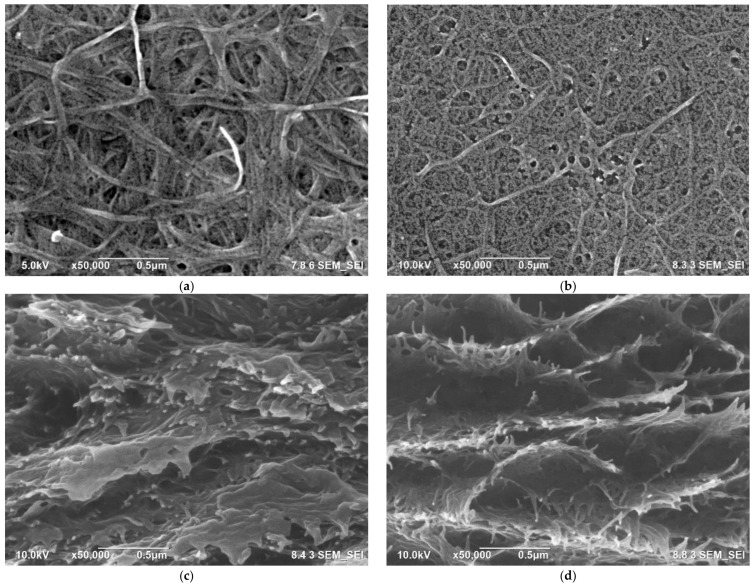

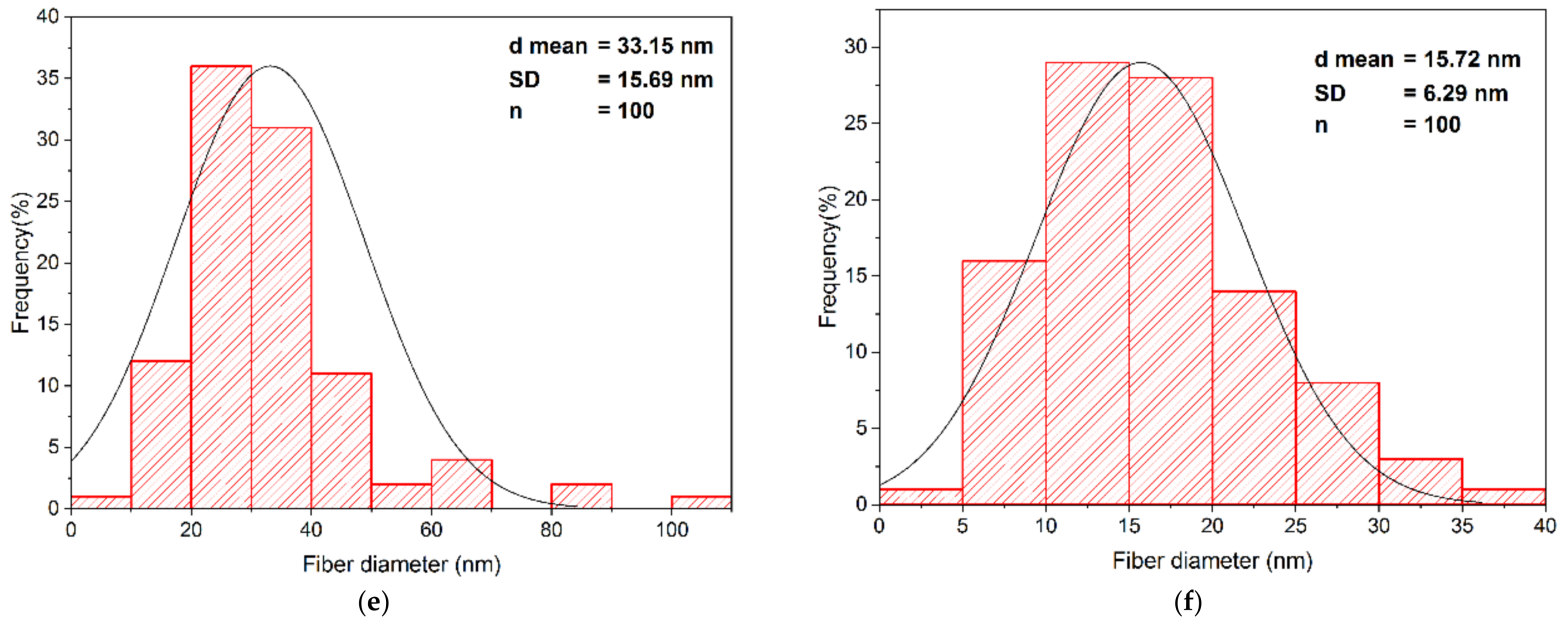
FESEM morphology in the surfaces of the UB-0 (**a**) and the UB-750 (**b**) and in the cross-sections of the UB-0 (**c**) and the UB-750 (**d**), and the average diameter distribution of nanofibers from Figure 2a (**e**) and Figure 2b (**f**).

Figure 1a shows the 200 mesh dry BC powders. The TOBC suspension of the powder treated without and with ultrasonication is shown in Figure 1b. The transparency of the suspension increased with an increase in the ultrasonication power. This result was because more light was transmitted through the suspension, probably containing a larger fraction of nanofibers with a smaller dimension and better dispersion [25,37]. After drying this suspension, BC films prepared with similar volumes of 0 W, 250 W, 500 W, and 750 W sonicated suspensions had thicknesses of about 32 μm, 29 μm, 32 μm, and 31 μm, respectively, and they showed different degrees of transparency. Figure 1c shows that the clouds behind the nonsonicated film could not be clearly observed. However, the transparency of the film increased, and the object behind the film became more clearly visible after prolonged ultrasonication duration. For example, compared to the UB-0, the 750 W sonicated film was far more transparent (Figure 1d). This UB-750 also had good bendability (Figure 1e). This result agreed with previous work showing that cellulose film was more transparent after ultrasonication [25]. Figure 1f shows the transmittance value of all films, demonstrating that higher ultrasonication power intensity increased the clarity of the samples. Ultrasonication at 250 W increased the light transmittance at 650 nm from 20.7% to 49.3% (Table 1). The 750 W sonicated film presented the highest transparency due to the slightest light scattering. This light was scattered slightly due to smooth surfaces and highly dense nanofiber structures (see Figure 2a,b) [38,39]. Additionally, the volume fraction of the well-aligned cellulose nanofibers and nanovoids could increase after ultrasonication [40,41]. Therefore, more light passed through the sonicated film than the nonsonicated film.

### 4.2. FESEM Morphology

Figure 2a,b displays the FESEM morphology of the surface of the UB-0 and the UB-750 films, respectively. The nonsonicated sample contained long, large-diameter fibers and bound BC fibers. The average fiber diameter of this UB-0 film was 33 nm (Figure 2e). After the ultrasonication of 750 W, the diameter of fiber decreased to 15 nm (Figure 2f), and the length became shorter. These decreased dimensions were because microjets and shock waves from ultrasonication reduced the diameter and sectioned the fibers into shorter lengths [25,37]. Hence, the sonicated film had more compact structures, and the sonicated BC nanofibers had a larger surface-area-to-volume ratio than the nonsonicated ones [42]. In addition, the number of nanopores also increased after ultrasonication. This finding is in agreement with previous works [12,43]. Meanwhile, Figure 2c,d shows the appearance of the fracture surface in the cross-section. The striking difference is the number of frayed fibers, which were more prevalent in the UB-750 film than the UB-0 film. This result is due to slippage between fibers, and lower energy is required to loosen individual UB-750 fibers than for crack propagation throughout the film.

### 4.3. FTIR Spectra

**Figure 3:**
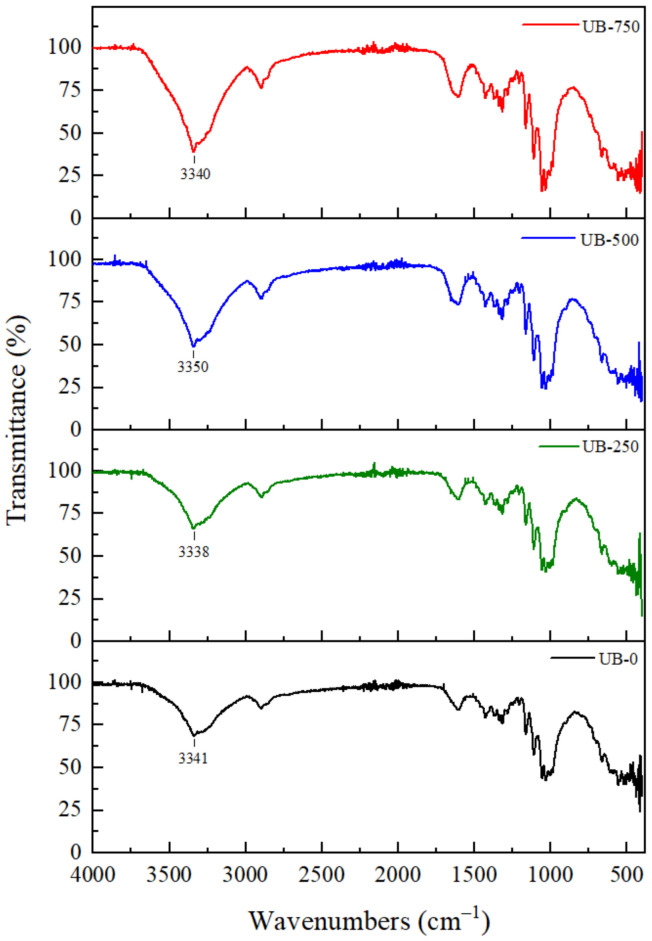
Effect of different ultrasonication powers on the FTIR spectrum.

**Figure 4:**
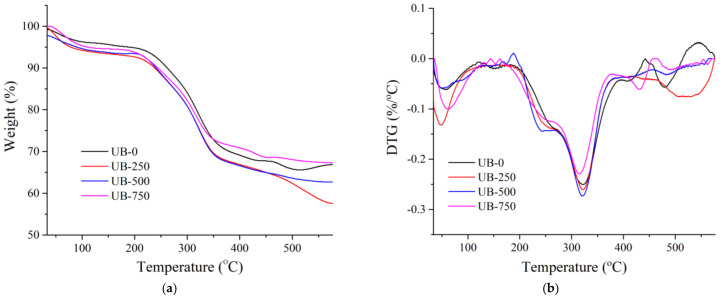
(**a**) Thermogravimetric (TGA) and (**b**) differential thermal analysis (DTA) curve for nonsonicated and sonicated BC film.

Figure 3 shows the FTIR spectra for BC films treated with different ultrasonication powers. The FTIR pattern of all films was broadly similar, indicating that ultrasonication treatment did not significantly affect the chemical structure of BC. This result is similar to previous findings [43]. However, the ultrasonication power of BC shifted the wavenumbers and intensities of the spectrum peaks. The shifting may be attributed to the changing cellulosic structure. The structural change was consistent with the XRD pattern shift (Figure 4). The UB-0 film presented the peak position of OH- stretching vibrations at 3341 cm^−1^. This position moved to 3338 cm^−1^ (UB-250 film), 3350 (UB-500 film), and 3340 cm^−1^ (UB-750 film) after the sonication of 250 W, 500 W, and 750 W, respectively. This shifted wavenumber may be due to increased hydrogen bond interlinking between the cellulose molecules [44]. In addition, the value of the peak intensity of these hydroxyl groups decreased after ultrasonication. For example, the intensity value for the UB-0 film was 70%, shifting to 40% for the UB-750 film. Similarly, the peaks corresponding to the -OH bending of adsorbed water at about 1600 cm^−1^ also decreased in intensity. This lower transmittance value may correspond to an increase in -OH vibrations as a result of more free -OH groups available in the polymer chains due to the smaller fiber size after the prolonged ultrasonication duration [25,45,46].

### 4.4. Thermal Properties

Figure 4 displays the thermal properties of BC without and with ultrasonication. The first weight losses of all BC samples (60–150 °C) were due to the evaporation of absorbed water [47]. The samples presented various weight losses because of the different amounts of water evaporated. In the second step, in the range of 300–420 °C, considerable weight loss is associated with the decomposition of cellulose [48]. After ultrasonication, the thermal resistance of the sample in this temperature range decreased. This result was evident from the maximum decomposition temperature (T_max_) and the latent heat of fusion. Table 2 shows the T_max_ and latent heat of fusion for all samples. T_max_ for the UB-0 sample was 301.3 °C; this decreased to 214.7 °C after ultrasonication at 750 W. The latent heat of fusion for the 750 W sonicated film was 501.6 J/g, significantly lower than the UB-0 film (532.8 J/g). This finding was probably because the increase in ultrasonic energy made the fiber size smaller, resulting in a higher specific surface area per unit volume (specific surface); consequently, it reacted at a much faster rate at lower degradation temperatures [37]. This phenomenon was similar to the results reported in a previous work that found that the depolymerization of the BC chain caused a decrease in thermal resistance. The smaller nanofiber size resulted in a decomposition temperature decrease [45]. Finally, a third weight loss was present over 420 °C due to the decomposition of charcoal.

### 4.5. Tensile Properties

**Figure 5:**
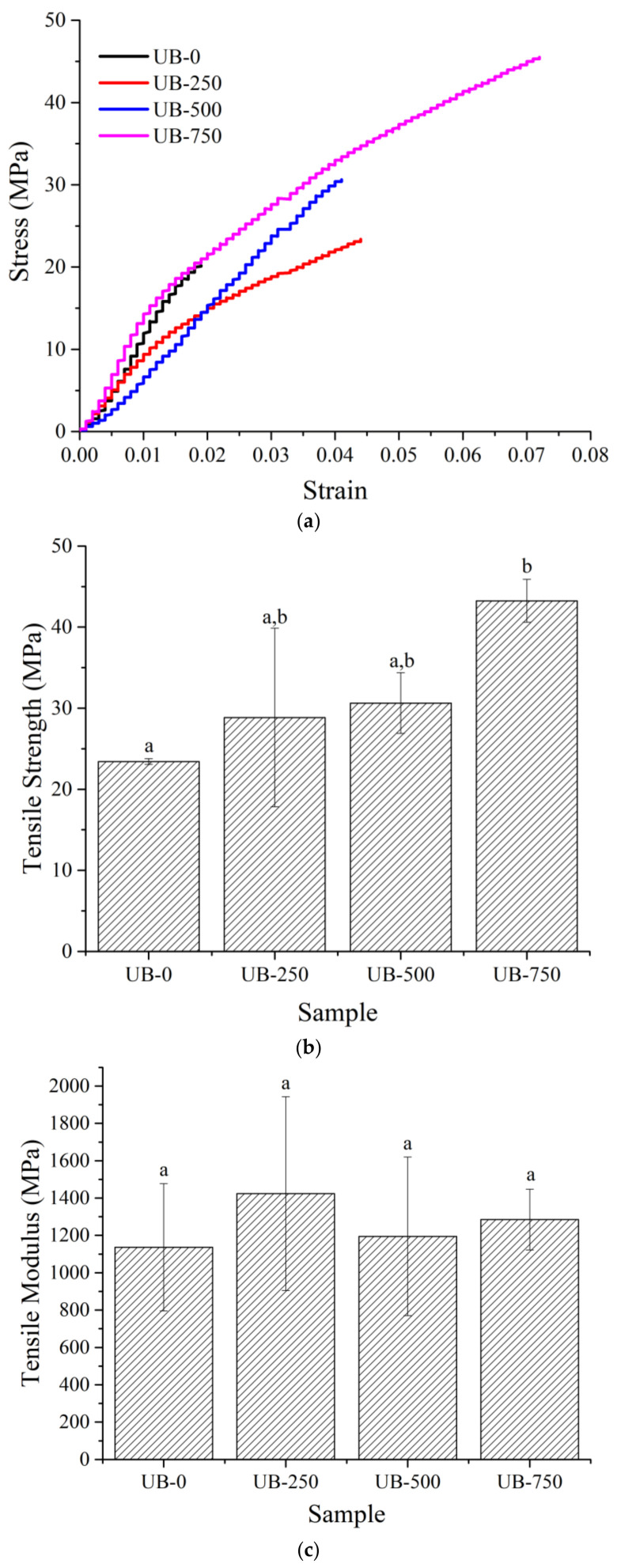

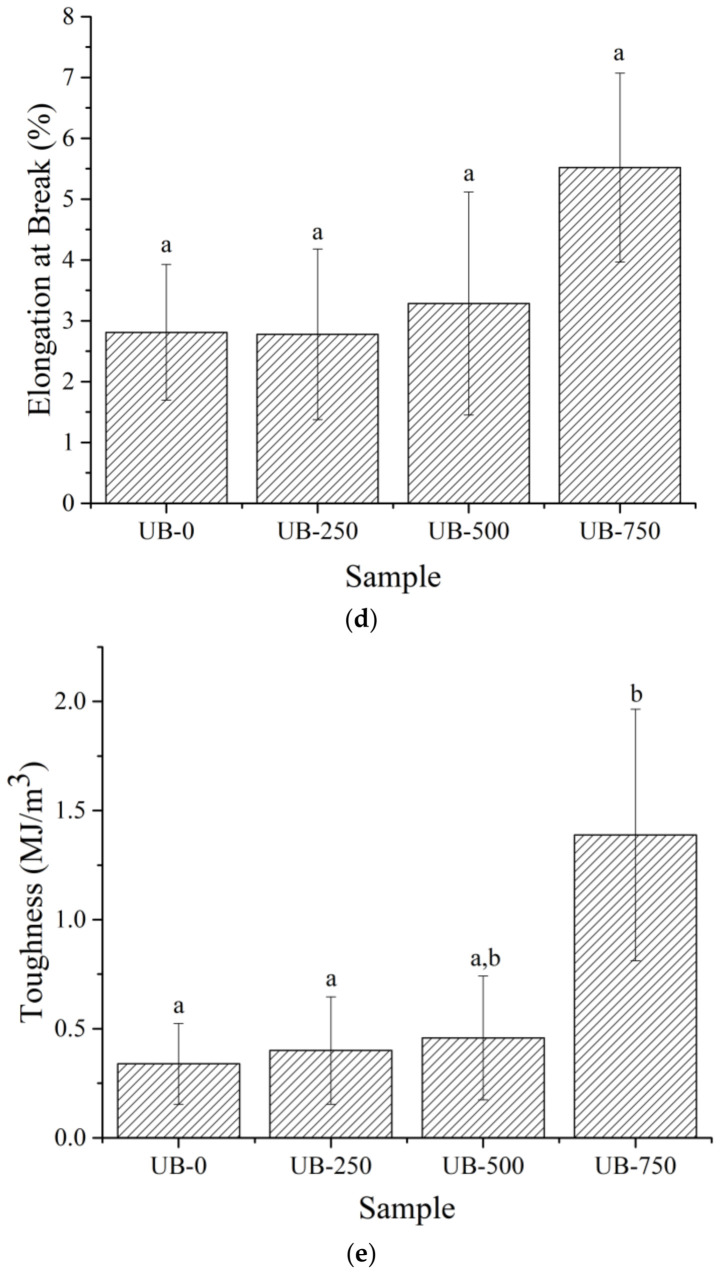
Stress–strain curve of nonsonicated and sonicated films (**a**). Average values of TS (**b**), TM (**c**), EB (**d**), and TN (**e**) for all tested films. Different letters (a,b) for each data point indicate a significant difference in mean values (*p* ≤ 0.05). The same letter indicates that values are not significantly different.

Figure 5a shows a uniaxial stress–strain curve of the samples. The TOBC film without ultrasonication presented a low stress–strain value due to the low compactness of the larger nonsonicated fibers (Figure 2a) compared to the smaller, finer ultrasonicated TOBC nanofibers (Figure 2b). This phenomenon was due to the weak surface bond between the fibers. After ultrasonication, tensile properties increased. Figure 5b–e shows the average value of tensile strength (TS), tensile modulus (TM), elongation at break (EB), and toughness (TN) for all films. The TM was not significantly affected by sonication. However, the TS, EB, and TN values were raised. These had values of 23.4 MPa, 2.8%, and 0.34 MJ/m^3^, respectively, for the nonsonicated film. With 750 W sonication, TS increased by 85% (43.2 MPa), EB by 96% (5.5%), and TN by 308% (1.39 MJ/m^3^), compared to the UB-0 film. This result was because the ultrasonication assisted in improving hydrogen bond interlinking and the compactness of the cellulose chains [40,49]. This finding was consistent with the higher compatibility (Figure 2d) and shifting of the peak position of OH functional groups at around 3341 cm^−1^ (Figure 3) after ultrasonication.

### 4.6. X-ray Diffraction

**Figure 6:**
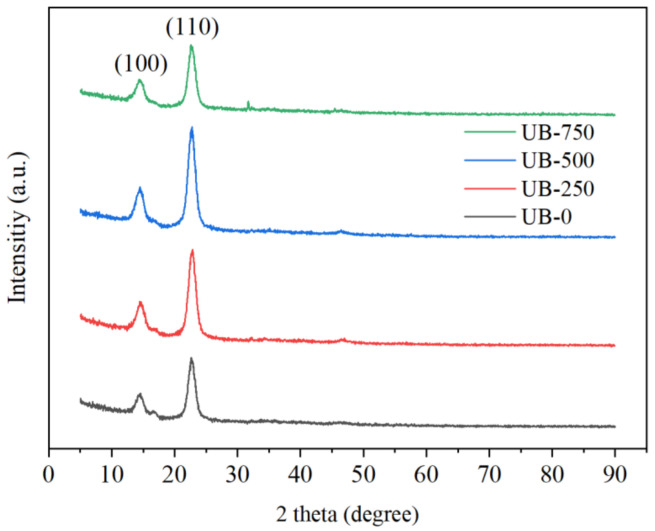
X-ray diffraction patterns of films.

Figure 6 shows the X-ray diffraction pattern of BC without and with ultrasonication treatment. All nonsonicated and sonicated BC films had typical cellulose Iα diffraction patterns with two main peaks at 2θ = 22° (110) and 14° (100). This result was consistent with the previously reported diffraction pattern [50]. After increasing the ultrasonication treatment, the CI shifted from 86.8% to 88.3% to 89.0%. This increased CI was due to the reduction in amorphous regions [51]. The kinetic energy of liquid jets destroyed the amorphous sections due to their lower resistance against sonication attacks compared to the more strongly bonded crystalline domains [52,53]. This phenomenon agreed with the higher transparency of the sonicated film than the nonsonicated one shown in Figure 1f. After ultrasonication, the *d*-spacing of the (200) plane tended to decrease, as illustrated by the UB-0, UB-500, and UB-750 data (Table 1). These shifts indicated that the sonicated BC film contained a more compact chain structure than the nonsonicated film [54]. This result was consistent with the stress–strain curve (Figure 5b), showing the higher tensile strength of the BC film after ultrasonication. Slippage was more difficult in the more strongly bonded, tightly packed cellulose chains of the sonicated film than in the nonsonicated film [55,56].

### 4.7. Electrical Properties

**Figure 7:**
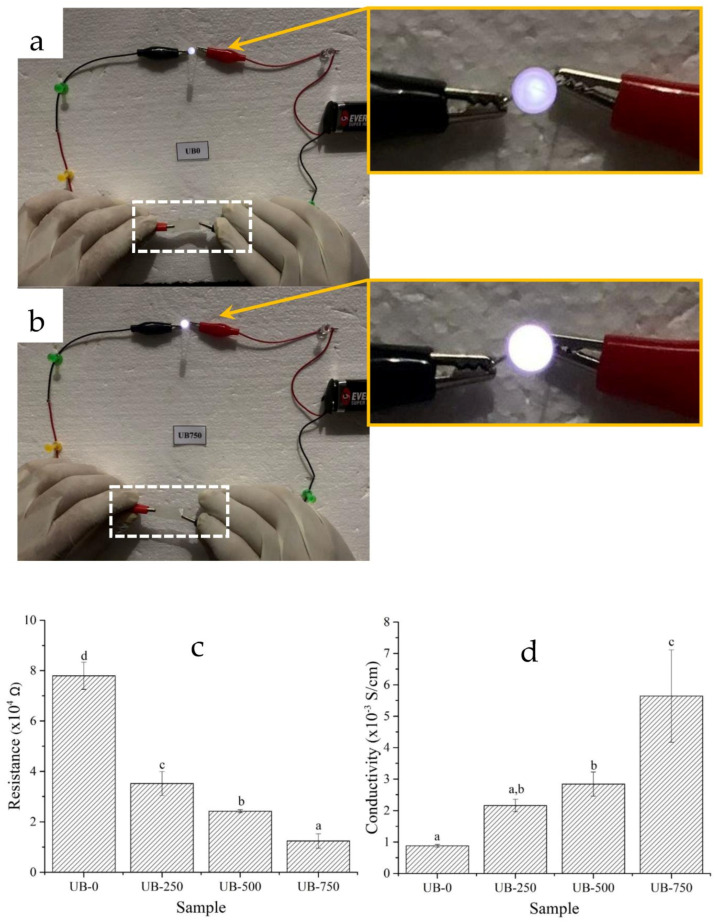
The light-emitting diodes (LED, 3 V) connected with UB-0 (**a**) and UB-750 (**b**) films with a thickness of 32 μm and 31 μm, respectively. The electrical resistance (**c**) and conductivity (**d**) values of the nonsonicated (UG-0) and sonicated (UG-250, UB-500, and UG-750) films at 50% RH. Different letters (a,b,c,d) for each data point indicate a significant difference in mean values (*p* ≤ 0.05).

Figure 7a,b shows the closed direct current (DC) circuits used with a light-emitting diode (LED) and UB-0 or UB-750 films (white dotted line box) to complete the circuit. After plugging a 9 V battery, a 3 V LED connected to the UB-750 film glowed brighter than that connected using UB-0 (solid orange lines). This result indicated that the UB-750 was a better electrical conductor than the UB-0. Figure 7c,d presents the average values of resistance and conductivity for all studied samples. From these figures, the UB-0 film had the highest resistance of 77.9 kΩ and the lowest conductivity of 0.088 S/m. This low electrical conductivity value could be due to the poor electron movement in the nonsonicated sample. With increasing ultrasonication power (250 W, 500 W, and 750 W), the electrical resistance of films reduced significantly (Figure 7c), and conductivity improved. From Figure 7d, the conductivity value of the UB-250, UB-500, and UB-750 films were 0.216 S/m, 0.284 S/m, and 0.564 S/m, respectively. The high ionic conductivity value may correspond to the increased number of charge carriers from the vibrating molecules of functional groups [57]. Molecules with stretching, bending, and twisting vibrations can help to move electric currents. This phenomenon agrees with the FTIR curve (Figure 3), showing the greater area under the FTIR curve of hydroxyl groups due to the higher intensity of the -OH stretching vibration of sonicated films than nonsonicated films. In addition, compared to the nonsonicated film, the sonicated film had more volume fractions of nanovoids and well-aligned short nanofibers (as shown in Figure 1), which promoted the more effortless mobility of free charges [31]. That is why sonicated films are more conductive than nonsonicated ones. Remarkably, this conductivity was higher than the one that the literature reported (0.005 S/m), which prepared BC films with 10% reduced graphene oxide and sonicated with the power of 100 W [58]. On the contrary, the literature discloses that sonicated BC in high amounts will fuse the neighboring BC fibrils, which leads to the increment in particle size or width of the fibrils value. This will decrease the conductivity because the volume fraction of nanovoids and the well-aligned short nanofibers will be less. This finding proves that ultrasonic power affects the electrical conductivity of the BC film if it is operated in optimal amounts [59].

## 5. Conclusions

This work prepared a pure cellulose film via ultrasonication and TEMPO treatment BC powders to increase its properties. The BC solution was sonicated with different powers (0, 250, 500, and 750 W). It was found that the sonicated BC film was more conductive electrically. Ultrasonication decreased the BC fiber size and increased the volume fraction of nanovoids, well-aligned short nanofibers, and free hydroxyl groups, facilitating greater ion mobility. Sonication at 750 W produced the TOBC film with the best electrical conductivity, transparency, and tensile strength. This finding of electrical conductivity was higher than the one in the literature, which prepared BC film with graphene oxide and low ultrasonic power. This suggests that ultrasonic power could affect or improve the properties of the BC film. However, the sonicated film presented a lower thermal resistance than the nonsonicated one. These results indicate that dry BC powder and ultrasonication have good potential as a base material and method for producing a conductive, transparent, strong, and flexible film potential for environmentally friendly electronic device applications.

## Figures and Tables

**Table 1:** Transmittance from Figure 1f, CI for different BC films.

Samples	Transmittance (%) at 650 nm	CI (%) of (200) Plane	*d*-Spacing (Å) of (200) Plane
UB-0	20.7	86.8	3.92
UB-250	49.3	88.3	3.95
UB-500	53.9	89.0	3.89
UB-750	56.6	86.2	3.85

**Table 2:** T_max_ and latent heat of fusion for different BC films.

Samples	T_max_ (°C)	Latent Heat of Fusion (J/g)
UB-0	301.3	532.79
UB-250	276.2	385.83
UB-500	288.9	433.26
UB-750	214.7	501.62

## Data Availability

The data presented in this study are available on request from the corresponding author.
